# Comparison of different selection traits for identification of phosphorus use efficient lines in mungbean

**DOI:** 10.7717/peerj.12156

**Published:** 2021-10-08

**Authors:** Venkata Ravi Prakash Reddy, Harsh Kumar Dikshit, Gyan Prakash Mishra, Muraleedhar Aski, Akanksha Singh, Ruchi Bansal, Renu Pandey, Ramakrishnan Madhavan Nair

**Affiliations:** 1 Division of Genetics, ICAR- Indian Agricultural Research Institute, New Delhi, India; 2 Acharya N.G. Ranga Agricultural University Regional Agricultural Research Station, Nandyal, India; 3 Amity Institute of Organic Agriculture, Amity University, Noida, Uttar Pradesh, India; 4 Division of Germplasm Evaluation, ICAR-National Bureau of Plant Genetic Resources, New Delhi, India; 5 Division of Plant Physiology, ICAR-Indian Agricultural Research Institute, New Delhi, India; 6 World Vegetable Center, South Asia/Central Asia, Patencheru, Hyderabad, Telangana, India

**Keywords:** Vigna radiata, Phosphorus, Biomass, Total phosphorus uptake, Phosphorus utilization, Stress tolerance index, Categorization of genotypes

## Abstract

Phosphorus (P) is one of the major constraints for crop growth and development, owing to low availability and least mobility in many tropical soil conditions. Categorization of existing germplasm under P deficient conditions is a prerequisite for the selection and development of P efficient genotypes in the mungbean. In the present investigation, 36 diverse genotypes were categorized for phosphorus use efficiency traits using four different techniques for identification of phosphorus use efficient mungbean genotypes. The studied genotypes were categorized for P efficiency based on efficiency, responsiveness, and stress tolerance score of genotypes under normal and low P conditions. The mean values of traits, root dry mass, root to shoot ratio, and P utilization efficiency are significantly higher under low P conditions indicating the high responsiveness of traits to P deficiency. The presence of significant interaction between genotypes and P treatment indicates the evaluated genotypes were significantly affected by P treatment for studied traits. The total P uptake showed significant and positive correlations with root dry mass, shoot dry mass, total dry mass,and P concentration under both P regimes. Out of the four techniques used for the categorization of genotypes for P efficiency, three techniques revealed that the genotype PUSA 1333, followed by Pusa Vishal, PUSA 1031, and Pusa Ratna is efficient. The categorization based on stress tolerance score is the finest way to study variation and for the selection of contrasting genotypes for P efficiency. The identified P efficient genotypes would be valuable resources for genetic enhancement of P use efficiency in mungbean breeding.

## Introduction

Mungbean is an important grain legume in Asia, with an area and production of 7.3 m ha and 5.3 m tonnes, respectively (Nair & Schreinemachers, 2020). The seeds of the mungbean are mainly used in making *dhal*, soup, sweets, dalmot, and noodles, (Lambrides & Godwin, 2007). Mungbean sprouts are a good source of nutrients like iron and zinc and are used as fresh salads in Southeast Asia (Tang et al., 2014). Mungbean seeds are rich in proteins (25–28%), carbohydrates (62–65%), fiber (4.5–5.5%), and oils (3.5–4.5%). It is a potential food supplement to alleviate malnutrition (Ganesan & Xu, 2018).

Phosphorus (P) is the second most important macronutrient following nitrogen (N) that is necessary for plant growth and development. It is an essential constituent of phospholipids, nucleic acids, and energy intermediates like ATP and NADPH in living cells (Heuer et al., 2017). It is essential for many physiological processes like seed germination, flowering, and fruit formation in crop plants (Panigrahy, Rao & Sarla, 2009). P deficiency in plants leads to activation of mechanisms like change in root morphological traits, increased expression of P transporters, higher root to shoot ratio, root organic acid exudation, and root microbial association (Lambers et al., 2006; Shen et al., 2011). Further P deficiency results in stunted growth with higher root biomass than shoot biomass in crop plants (Kim & Li, 2016). In soils, P deficiency can be addressed by the use of P-containing inorganic fertilizers. The main source of inorganic P is rock phosphate, which is minable only in a few countries of the world (Cordell & White, 2013). By anticipating future shortages, countries like China and the USA have stopped export to other countries in the world for strategic reasons (Van de Wiel, van der Linden & Scholten, 2016). This calls for the development of cultivars with high P use efficiency (PUE) in crop plants.

PUE is well-defined as the capacity to produce higher biomass/yield per unit P taken up by the plant (Hammond et al., 2009). PUE is a multifarious trait and can be distinguished into two important mechanisms *i.e.,* P uptake efficiency (PUpE) and P utilization efficiency (PUtE) (Wang, Shen & Liao, 2010). Thus, PUE depends on the ability of P uptake and its utilization in biomass production by crop plants (Van de Wiel, van der Linden & Scholten, 2016). The exploitation of root and shoot biomass traits, total P uptake (TPU), and PUtE traits will provide the way for the enhancement of PUE in crop plants. For various measures of PUE, genetic variation among genotypes was observed in rice (Irfan et al., 2019), wheat (Deng et al., 2018), maize (Jiang et al., 2019), soybean (Furlani et al., 2002; Zhou et al., 2016), common bean (Shanka et al., 2018), mungbean (Irfan, Shah & Abbas, 2017), Brassica (Akhtar et al., 2007) and cotton (Iqbal et al., 2019). However, for measuring the PUE of genotypes, attention should be given to comparing the genetic and physiological traits of crop plants. Testing of genotypes with different methods in the same environment will help to identify differences in P efficiency. Ma (2000) and Hinsinger et al. (2011) focused mainly on root traits for measuring PUE in crop plants. However, both root and shoot traits are important for P acquisition and internal utilization respectively. Thus, screening and categorization of genotypes based on different selection traits is a prerequisite for the identification of P efficient genotypes in varying P regimes (Abbas et al., 2018; Irfan et al., 2020).

Under low P input conditions, P efficient genotypes with high biomass production at low P condition are desirable, while P responsive genotypes which can produce more biomass with the application of P fertilizer are preferable under a high input system (Yaseen & Malhi, 2009). For the categorization of genotypes, different scientists proposed various methods with some advantages and disadvantages under the P deficient condition. According to Bilal et al. (2018), the genotypes may be categorized into three classes namely efficient, medium and inefficient types based onthe scoring of genotypes for biomass, P uptake, and utilization traits under normal and low P conditions. Based on efficiency and responsiveness of genotypes to varying P conditions, they can be categorized into efficient and responsive, efficient and non-responsive, inefficient and responsive and inefficient and non-responsive types (Gerloff, 1977). Further, to study the significant variation between the genotypes for P efficiency and responsiveness, the genotypes can be categorized into high, medium, and low groups based on P uptake and total biomass traits (Gill et al., 2004). Genotypes can also be categorized based on the stress tolerance score calculated for total biomass for each genotype (Thiry et al., 2016). Therefore, the efficiency of genotype under the P limiting condition differs with the parameters and P efficiency calculation methods (Aziz et al., 2014; Sanadana & Pinochet, 2016). Besides, a well understanding of different parameters and methods of P efficiency calculation is required for the incorporation of P efficiency in assessing genotypes. This study aimed to identify phosphorus use efficient genotypes of mungbean comparing four categorization methods (using different parameters and indices.

## Materials and Methods

The present study was carried out with the 36 diverse mungbean genotypes to identify the P use efficient ones at the seedling stage (Table S1). The study was conducted in a hydroponic system at a controlled greenhouse condition at Indian Agricultural Research Station (IARI), New Delhi. The weather conditions maintained in the greenhouse were 30°/18 °C day/night temperature, 12 h photoperiod, and 90% relative humidity. The seeds of all 36 genotypes were washed with 0.1% (w/v) mercuric chloride and kept for germination. After the appearance of cotyledonary leaves, the evenly germinated seedlings were shifted to hydroponic trays containing modified Hoagland solution (Sivasakthi et al., 2017). The control and treatment conditions for P stress were maintained with two levels of P *i.e.,* normal P (NP) (250 µM) and low P (LP) (3 µM) (Reddy et al., 2020). The nutrient solution was changed every alternate day and the pH of the nutrient solution was continued around 6.0 using 1M HCL and 1M KOH solution.

The 21 days grown seedlings under both normal and low P regimes were removed and dried at 60 °C until constant mass for measuring the biomass and P efficiency traits was obtained. The separated root and shoot portions were used for measuring the root dry mass (RDM) (g/plant) and shoot dry mass (SDM) (g/plant), respectively using precision weighing balance. The root and shoot dry mass of each plant were summed and divided to get the total dry mass(TDM) (g/plant) and root to shoot ratio (RSR), respectively. The dried root and shoot portions were mixed and ground to obtain a fine powder for estimating the P concentration (PC) (mg P/g dry mass). The P content of the sample was estimated by digesting the sample with a di-acid mixture (9:4 ratio of nitric acid and perchloric acid) followed bythe calorimetric method given by Murphy & Riley (1962). The total P uptake (TPU) (mg P/plant) by the plant was obtained by multiplying the TDM with PC (Wang et al., 2017). The P utilization efficiency (PUtE) under both OP and LP conditions was calculated by using the formula (Wang et al., 2017):

PUtE (g dry mass/mg P) = Total dry mass/ total P uptake by plant

The seedlings in the present investigation were assessed in a completely randomized design (CRD) with three replications per genotype. The obtained data from genotypes grown in NP and LP regimes were statistically analyzed by STAR (Statistical Tool for Agricultural Research) 2.1.0 software (Gulles et al., 2014). Further, the 36 genotypes were categorized based on different physiological and genetic traits for the identification of P efficient genotypes under normal and low phosphorus conditions. The four techniques used for the categorization of genotypes are as follows

**Technique 1**: As per the method given by Osborne & Rengel (2002) and Aziz et al. (2011), the mungbean genotypes were classified into efficient (E), medium (M), and inefficient (I) types based on the absolute values assigned to each genotype using population mean (µ) and standard deviation (SD) of each parameter under both P regimes. The mean value of efficient genotype was >µ+SD, for medium, it was ranging between µ+SD to µ-SD, and for inefficient, it was <µ-SD. The score assigned to efficient, medium, and inefficient genotypes is 3, 2, and 1, respectively. Further, the distinct scores of each parameters were summed to get the cumulative score of the respective genotype.

**Technique 2**: According to Gerhardt et al. (2017), the genotypes can be categorized for efficiency and responsiveness of genotypes to P supply based on the dry matter production and P efficiency of genotypes under respective P environments. The efficient genotype produced higher total dry mass as compared to the average total dry mass of studied genotypes and responsive genotypes exhibit higher PUtE in comparison to average PUtE. The studied genotypes were delineated in four groups (i) efficient and non-responsive (ENR), ii) efficient and responsive (ER) iii) inefficient and non-responsive (INR) (iv) inefficient but responsive (IR) as suggested (Fageria, 1993; Kosar et al., 2003).

**Technique 3**: According to Gill et al. (2004), the genotypes can be categorized into nine groups by developing ordinary plots representing TDM and TPU on the *x*-axis and *y*-axis, respectively. The studied genotypes were classified in nine groups viz., high dry mass-high P (HDM-HP), high dry mass-medium P (HDM-MP), high dry mass-low P (HDM-LP), medium-dry mass-high P (MDM-HP),), medium-dry mass-medium P (MDM-MP), medium-dry mass-low P (MDM-LP, low dry mass-high P (LDM-HP),), low dry mass-medium P 165 (LDM-MP) and low dry mass-low P (LDM-LP).The mean value of genotype is >µ+SD, it was assigned as high, medium type with performance between µ+SD to µ-SD and low with performance <µ-SD. Where, µ and SD are the population’s mean and standard deviation, respectively. The ordination plots for the categorization of cultivars were developed with the MS-EXCEL program.

**Technique 4**: The P deficiency tolerance indices were calculated based on the TDM for all genotypes by following the equations given by Negarestani et al. (2019) and Grzesiak et al. (2019).

\begin{eqnarray*}& & \text{Stress susceptibility index (SSI)}=(1-T/C)/(1-xT/xC) \end{eqnarray*}

\begin{eqnarray*}& & \text{Mean productivity index (MPI)}=(C+T)/2 \end{eqnarray*}

\begin{eqnarray*}& & \text{Geometric mean productivity index (GMPI)}=\sqrt{C\times T}\sqrt{C\times T} \end{eqnarray*}

\begin{eqnarray*}& & \text{Harmonic mean index (HMI)}=2(C\times T)/(C+T) \end{eqnarray*}

\begin{eqnarray*}& & \text{Stress tolerance index (STI)}=(C\times T)/(xC)^{\text{2}} \end{eqnarray*}

\begin{eqnarray*}& & \text{Tolerance index (TI)}=C-T \end{eqnarray*}

\begin{eqnarray*}& & \text{Stress index (SI)}=T/C \end{eqnarray*}

Where, C and T represent the total dry mass (TDM) of genotypes under control and treatment conditions, respectively. *x*C and *x*T represent the average total dry mass (TDM) of all studied genotypes under control and treatment conditions, respectively.

Further, the standardized values of all indices were used to calculate the stress tolerance score of all genotypes grown under NP and LP conditions by following the equation (Thiry et al., 2016; Negarestani et al., 2019).

\begin{eqnarray*}\text{Stress tolerance score}(STC)=SSI+MPI+GMPI+HMI+STI+TI+SI. \end{eqnarray*}

## Results

### Descriptive statistics and analysis of variance of measured traits

The descriptive statistics of seven measured traits as explanatory variables of PUE among 36 studied genotypes grown in NP and LP conditions are presented in Table 1. The mean values of SDM, TDM, PC, and TPU were higher in the NP condition compared to the LP condition. While the mean values of RDM, RSR, and PUtE were much higher under LP conditions. The highest percentage reduction was noticed for TPU (−79.13) followed by PC (−72.26) under LP condition compared to NP condition. Whereas the highest gain was noticed for PUtE (317.78) followed by RSR (64.62) under LP condition. Among the 36 genotypes, the TPU ranged from 0.717 to 4.793 mg P/plant and 0.115 to 0.822 mg P/plant under NP and LP conditions, respectively, whereas PUtE ranged from 0.091 to 0.187 g dry mass/mg P and 0.279 to 1.557 g dry mass/mg P under NP and LP conditions, respectively among the tested genotypes.The analysis of variance of measured traits revealed significant variation among genotypes studied in the experiment (Table 2).There was a significant interaction between genotype and P treatment. The coefficient of variation ranged from 11.69% (TDM) to 37.09% (PUtE) among the traits investigated. The highest and lowest broad-sense heritability was noticed for RDM (0.96) and PUtE (0.23), respectively among the traits.

**Table 1:** Descriptive statistics of the measured traits in mungbean genotypes under normal and low phosphorus conditions.

Trait	P level	Mean	Minimum	Maximum	SD	SE	Reduction (%)
RDM	NP	0.037	0.015	0.110	0.021	0.003	10.81
	LP	0.041	0.013	0.107	0.019	0.003	
SDM	NP	0.183	0.087	0.437	0.060	0.010	−31.70
	LP	0.125	0.077	0.190	0.023	0.004	
RSR	NP	0.195	0.092	0.312	0.062	0.010	64.62
	LP	0.321	0.179	0.563	0.107	0.018	
TDM	NP	0.220	0.107	0.547	0.078	0.013	−24.55
	LP	0.166	0.090	0.297	0.039	0.007	
PC	NP	7.640	5.355	11.055	1.265	0.211	−72.26
	LP	2.119	0.675	3.705	0.623	0.104	
TPU	NP	1.706	0.717	4.793	0.774	0.129	−79.13
	LP	0.356	0.115	0.822	0.156	0.026	
PUtE	NP	0.135	0.091	0.187	0.021	0.004	317.78
	LP	0.564	0.279	1.557	0.284	0.047	

**Notes.**

RDM root dry mass SDM shoot dry mass RSR root to shoot ratio TDM total dry mass PC phosphorus concentration TPU total phosphorus uptake PUtE phosphorus utilization efficiency NP normal phosphorus LP low phosphorus SD Standard deviation SE Standard error

**Table 2:** Analysis of variance for the measured traits under normal and low phosphorus conditions.

Variables	Mean sum of squares			F value			*P* value significance			CV (%)	Heritability
	G	P	G × P	G	P	G × P	G	P	G × P		
df	35	1	35	35	1	35	35	1	35		
RDM	0.002	0.001	0.000	83.30	38.18	5.31	***	***	***	13.46	0.96
SDM	0.009	0.180	0.003	23.31	467.22	8.76	***	***	***	12.72	0.62
RSR	0.037	0.861	0.009	24.87	573.82	5.87	***	***	***	15.03	0.76
TDM	0.019	0.154	0.004	36.97	301.55	7.98	***	***	***	11.69	0.78
PC	3.607	1645.898	2.354	8.34	3804.42	2.354	***	***	***	13.48	0.42
TPU	1.225	98.505	0.645	23.75	1909.65	12.51	***	***	***	22.03	0.51
PUtE	0.127	9.934	0.116	2.79	217.75	2.55	***	***	**	37.09	0.23

**Notes.**

G genotype P phenotype G ×P genotype × phosphorus interaction CV coefficient of variation RDM root dry mass SDM shoot dry mass RSR root to shoot ratio TDM total dry mass PC phosphorus concentration TPU total phosphorus uptake PUtE phosphorus utilization efficiency

**Significance at *P* < 0.01.

***Significance at *P* < 0.001.

### Correlation coefficients between measured traits

The Pearson’s correlation coefficients between measured traits studied in NP and LP regimes are presented in Table 3. The highest positive correlation was noticed between TDM and SDM under both NP (0.989) and LP (0.941) conditions. The measured trait TPU showed significant and positive correlation with RDM, SDM, RSR, TDM and PC under both NP (*r* values of 0.862, 0.919, 0.407, 0.935 and 0.571, respectively) and LP (*r* values of 0.739, 0.584, 0.632, 0.705 and 0.798, respectively) conditions. The PUtE showed a significant and negative correlation with PC and TPU under NP (*r* values of −0.965 and −0.538, respectively) and LP (*r* values of −0.814 and −0.601, respectively) conditions. The traits RDM and TDM exhibited a significant and positive correlation between each other and with SDM, RSR, and TPU under both P conditions. The traits SDM and RSR showed a significant and positive correlation with RDM, TDM, and TPU under NP conditions. Whereas in LP condition, SDM and RSR showed a significant and positive correlation with each other and with RDM, RSR, TDM, and TPU. The trait PC exhibited significant positive and negative correlations with TPU and PUtE respectively in both NP and LP conditions.

**Table 3:** Pearson’s correlation coefficients between measured traits under normal P (NP) and low P (LP) conditions.

NP/LP	RDM	SDM	RSR	TDM	PC	TPU	PUtE
RDM	1.000	0.829^**^	0.757^**^	0.904^**^	0.253	0.862^**^	−0.224
SDM	0.715^**^	1.000	0.282	0.989^**^	0.249	0.919^**^	−0.238
RSR	0.913^**^	0.405^*^	1.000	0.420^*^	0.112	0.407^*^	−0.093
TDM	0.910^**^	0.941^**^	0.683^**^	1.000	0.258	0.935^**^	−0.242
PC	0.277	0.036	0.325	0.155	1.000	0.571^**^	−0.965^**^
TPU	0.739^**^	0.584^**^	0.632^**^	0.705^**^	0.798^**^	1.000	−0.538^**^
PUtE	−0.169	−0.028	−0.196	−0.099	−0.814^**^	−0.601^**^	1.000

**Notes.**

RDM root dry mass SDM shoot dry mass RSR root to shoot ratio TDM total dry mass PC phosphorus concentration TPU total phosphorus uptake PUtE phosphorus utilization efficiency

*Significance at *P* < 0.05.

**Significance at *P* < 0.01.

### Categorization of mungbean genotypes for PUE

**Technique 1:** The studied 36 genotypes showed significant differences for the traits RDM, SDM, RSR, TPU, and PUtE considered for scoring in both NP and LP regimes (Table 4). The genotypes PUSA 1333, Pusa Ratna, and Pusa Vishal recorded the highest score (13 out of 15) under LP conditions. Whereas under LP condition, PUSA 1333 (14 out of 15) followed by Pusa 1031 and Pusa Ratna (13 out of 15) recorded the highest score. The genotypes IC 282094, M 1443, and V 6183 (8 out of 15) under NP condition and IC 282094 and M 1443 (7 out of 15) under LP condition recorded the lowest score among the 36 genotypes. For overall performance among the studied genotypes, PUSA 1333 (27 out of 30) followed by Pusa Ratna (26 out of 30) recorded the highest score for PUE by summing up the score at both P levels. While the lowest score was recorded by IC 282094 and M 1443 (15 out of 30) indicating the poor performance for PUE among the genotypes. The poor performance of genotypes is mainly due to the low root, shoot biomass production, and total P uptake of genotypes under normal and low P conditions.

**Table 4:** Classification of genotypes into efficient (E), medium (M) and inefficient (i) types based on five traits recorded under normal and low phosphorus conditions.

Genotypes	Normal Phosphorus						Low phosphorus						Total score/ out of 30
	RDM	SDM	RSR	TPU	PUtE	Total score/ out of 15	RDM	SDM	RSR	TPU	PUtE	Total score/ out of 15	
EC 520029	M	M	M	M	M	10	M	I	M	M	M	9	19
EC 550851	M	M	I	M	I	8	M	I	M	M	M	9	17
GANGA 1	M	M	M	M	M	10	M	M	M	M	M	10	20
IC 282094	M	I	M	I	M	8	I	I	I	M	M	7	15
IPM 02-17	M	M	M	M	E	11	E	M	E	M	M	12	23
IPM 02-3	M	M	M	M	E	11	E	M	E	M	M	12	23
IPM 205-4	M	M	M	M	M	10	M	M	M	M	M	10	20
KM 16-69	M	M	I	M	M	9	M	E	M	M	M	11	20
KM 16-80	M	M	M	M	M	10	M	M	M	M	M	10	20
LGG 460	M	M	M	M	M	10	M	M	M	M	M	10	20
M 1032	M	M	M	M	M	10	M	M	M	M	M	10	20
M 1129	M	M	I	M	M	9	M	M	I	M	E	10	19
M 1209	M	M	M	M	E	11	M	M	I	M	M	9	20
M 1316	M	I	M	M	M	9	M	I	M	M	M	9	18
M 1443	I	M	I	I	E	8	I	I	I	I	E	7	15
M 209	M	I	M	M	M	9	M	M	M	M	M	10	19
M 512	M	M	M	M	M	10	M	I	M	M	M	9	19
M 961	M	M	M	M	M	10	M	M	M	M	M	10	20
MH 810	M	M	M	M	I	9	M	M	M	M	M	10	19
MH 934	M	M	M	M	M	10	M	M	M	M	M	10	20
ML 1451	M	M	M	M	M	10	M	M	M	M	M	10	20
ML 1666	M	M	M	M	M	10	M	M	M	I	E	10	20
ML 818	M	M	E	M	M	11	E	M	E	M	M	12	23
MUSKAN	M	M	M	M	M	10	M	M	M	M	M	10	20
PDM 139	E	M	E	M	M	12	E	M	M	M	M	11	23
PLM 167	M	M	M	M	M	10	M	M	M	E	M	11	21
PUSA 1031	E	M	E	E	I	12	E	E	M	E	M	13	25
PUSA 1132	M	M	M	M	M	10	M	M	M	M	M	10	20
PUSA 1333	E	E	M	E	M	13	E	E	E	E	M	14	27
Pusa Baisakhi	M	M	I	M	M	9	M	M	M	M	M	10	19
PusaRatna	E	M	E	E	M	13	E	M	E	E	M	13	26
Pusa Vishal	E	E	E	E	I	13	E	M	E	E	I	12	25
RMG 1028	M	M	M	M	M	10	M	I	M	M	M	9	19
RMG 1087	M	M	M	M	E	11	M	M	M	M	M	10	21
V 04-04	M	M	M	M	M	10	M	M	M	M	M	10	20
V 6183	M	M	I	M	I	8	M	M	M	M	M	10	18

**Notes.**

RDM root dry mass SDM shoot dry mass RSR root to shoot ratio TPU total phosphorus uptake PUtE phosphorus utilization efficiency E efficient M medium efficient I inefficient

**Figure 1:**
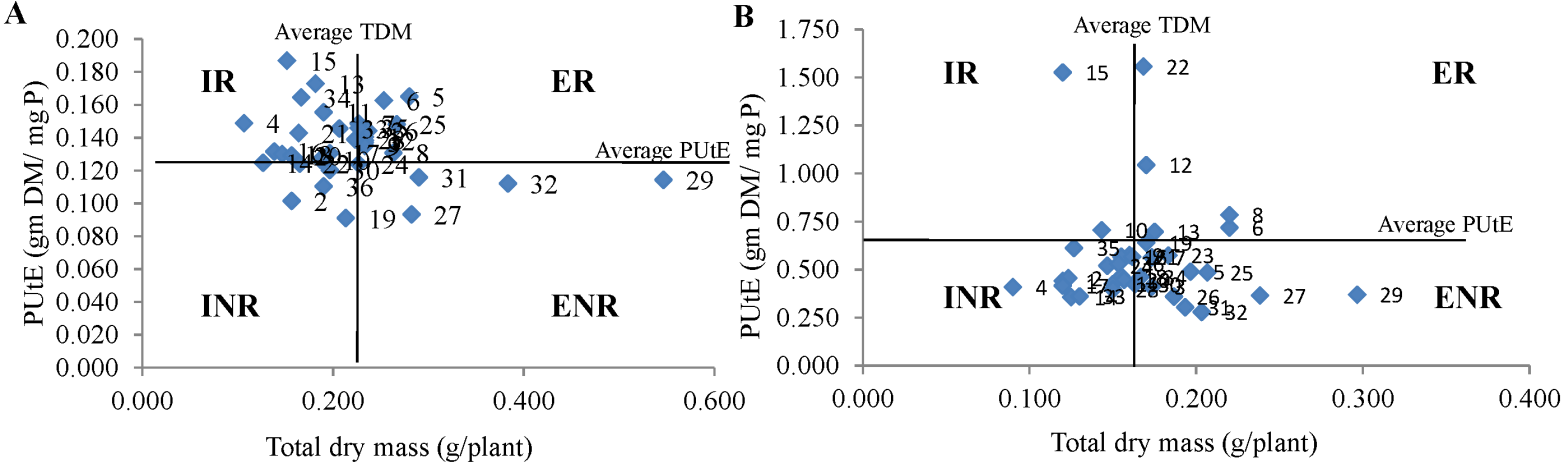
Classification of genotypes based on P utilization efficiency (PUtE) and total dry mass (TDM) at (A) normal P and (B) low P levels. This classification divides genotypes into four groups *i.e.,* efficient and responsive (ER), inefficient and responsive (IR), efficient and non responsive (ENR) and inefficient and non responsive (INR).

**Technique 2:** The genotypes studied in the experiment were categorized into four groups based on TDM and PUtE in both NP and LP regimes (Fig. 1). The genotypes IPM 02-17, IPM 02-3, IPM 205-4, KM 16-69, KM 16-80, M 1129, PDM 139, PUSA 1132, RMG 1028 and V 04-04 under NP condition and genotypes IPM 02-3, KM 16-69, M 1129, M 1209, MH 810 and ML 1666 under LP condition were classified in ER group. Further, the genotypes EC 550851, LGG 460, M 1316, MH 810, ML 1666, Pusa Baisakhi, and V 6183 were grouped in the INR category under NP condition. Whereas under LP condition, the genotypes EC 520029, EC 550851, IC 282094, M 1032, M 1316, M 512, MH 934, Muskan, PUSA 1132, RMG 1028, V 04-04 were categorized in the INR group. Interestingly the genotypes IPM 02-3, KM 16-69, and M 1129 were found in ER group under both P conditions. While the genotypes EC 550851 and M 1316 were categorized in the INR group under both P conditions. The genotypes PUSA 1031, PUSA 1333, Pusa Ratna, and Pusa Vishal were categorized in the ENR group under both P conditions. The single genotype M 1443 was categorized in the IR group under both P conditions.

**Figure 2:**
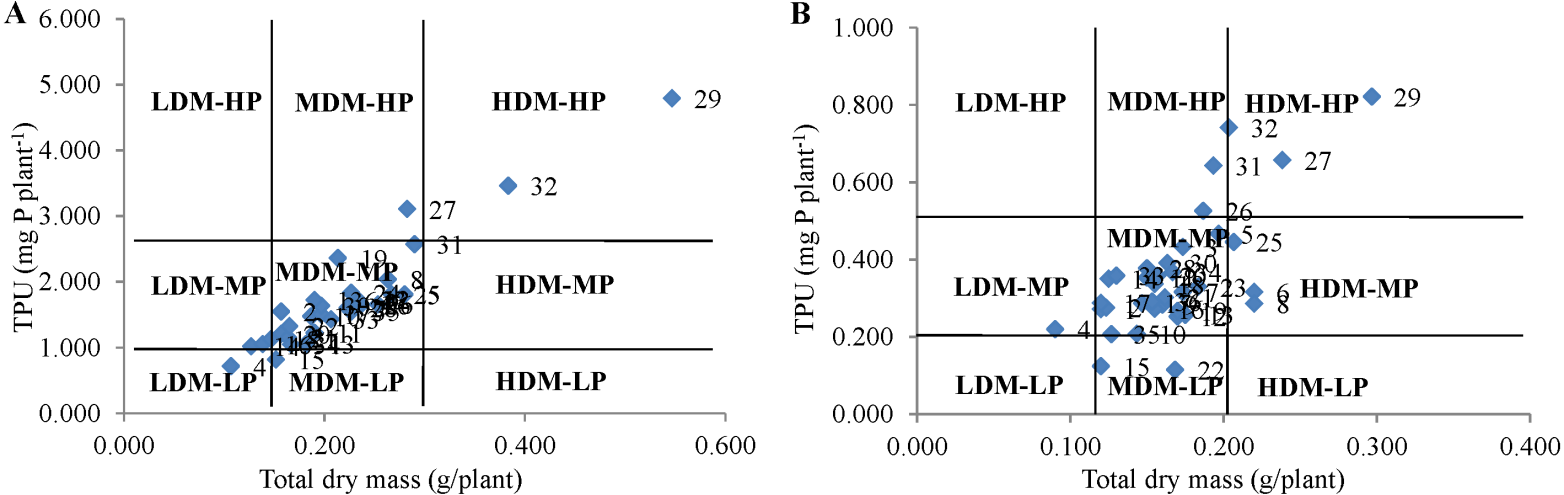
Classification of genotypes based on total P uptake (TPU) and total dry mass (TDM) at (A) normal P and (B) low P levels. This classification divides genotypes into nine groups viz., low dry mass-medium P (LDM-MP) and low dry mass-low P (LDM-LP), low dry mass-high P (LDM-HP). medium dry mass-medium P (MDM-MP), medium dry mass-low P (MDM-LP), medium dry mass-high P (MDM-HP), high dry mass-medium P (HDM-MP), high dry mass-low P (HDM-LP), high dry mass-high P (HDM-HP).

**Technique 3:** The categorization based on this method results in studying minor differences among the genotypes under both P conditions. The genotypes PUSA 1333 and Pusa Vishal under NP condition and PUSA 1031 and PUSA 1333 under LP condition were categorized under the HDM-HP group (Fig. 2). The genotype IC 282094 was categorized in LDM-LP and LDM-MP groups under NP and LP conditions, respectively. The genotypes IPM 02-3, KM 16-69, and PDM 139 were categorized in MDM-MP and HDM-MP groups in both NP and LP regimes, respectively. The majority of the genotypes were categorized in the MDM-MP group under both P conditions. While, none of the genotypes were categorized in LDM-HP, HDM-MP, and HDM-LP groups under NP conditions and LDM-HP, LDM-LP, and HDM-LP groups under LP condition. Interestingly the genotype PUSA 1333 with high TDM and TPU was categorized in the HDM-HP group under both NP and LP conditions.

**Table 5:** Phosphorus deficiency tolerance indices calculated for 36 genotypes grown under normal and low phosphorus conditions.

Genotypes	Phosphorus deficiency tolerance indices							
	SSI	MPI	GMPI	HMI	STI	TI	SI	STS
EC 520029	1.979	0.177	0.167	0.158	0.579	0.113	0.514	−2.355
EC 550851	0.867	0.140	0.139	0.138	0.399	0.033	0.787	−4.049
GANGA 1	1.048	0.203	0.201	0.199	0.836	0.060	0.743	0.486
IC 282094	0.637	0.098	0.098	0.098	0.198	0.017	0.844	−6.688
IPM 02-17	1.213	0.238	0.235	0.231	1.138	0.083	0.702	3.124
IPM 02-3	0.536	0.237	0.236	0.235	1.152	0.033	0.868	3.349
IPM 205-4	0.959	0.200	0.198	0.196	0.812	0.053	0.765	0.293
KM 16-69	0.670	0.242	0.241	0.240	1.197	0.043	0.835	3.671
KM 16-80	1.280	0.197	0.193	0.190	0.771	0.073	0.686	−0.193
LGG 460	0.946	0.165	0.164	0.162	0.553	0.043	0.768	−2.313
M 1032	0.858	0.170	0.169	0.168	0.589	0.040	0.789	−1.873
M 1129	1.106	0.202	0.199	0.197	0.820	0.063	0.729	0.318
M 1209	0.150	0.178	0.178	0.178	0.657	0.007	0.963	−0.726
M 1316	0.054	0.126	0.126	0.126	0.327	0.002	0.987	−4.240
M 1443	0.851	0.136	0.135	0.134	0.376	0.032	0.791	−4.329
M 209	−0.491	0.147	0.146	0.146	0.443	−0.017	1.120	−2.352
M 512	1.588	0.158	0.154	0.149	0.488	0.077	0.610	−3.378
M 961	−0.231	0.151	0.151	0.151	0.470	−0.008	1.057	−2.309
MH 810	0.828	0.192	0.190	0.189	0.749	0.043	0.797	−0.245
MH 934	0.000	0.157	0.157	0.157	0.507	0.000	1.000	−2.115
ML 1451	0.058	0.163	0.163	0.163	0.548	0.002	0.986	−1.739
ML 1666	−0.082	0.167	0.167	0.167	0.574	−0.003	1.020	−1.361
ML 818	0.873	0.208	0.207	0.205	0.884	0.050	0.786	0.985
MUSKAN	1.438	0.187	0.182	0.178	0.687	0.080	0.647	−1.087
PDM 139	0.917	0.237	0.235	0.233	1.139	0.060	0.775	3.156
PLM 167	0.861	0.212	0.210	0.209	0.913	0.050	0.789	1.248
PUSA 1031	0.635	0.260	0.259	0.258	1.390	0.044	0.844	5.155
PUSA 1132	1.338	0.187	0.183	0.179	0.692	0.073	0.672	−1.006
PUSA 1333	1.863	0.422	0.403	0.385	3.351	0.250	0.543	18.873
Pusa Baisakhi	0.691	0.180	0.179	0.178	0.664	0.033	0.831	−1.010
PusaRatna	1.358	0.242	0.237	0.232	1.158	0.097	0.667	3.306
Pusa Vishal	1.913	0.293	0.279	0.266	1.610	0.180	0.530	7.250
RMG 1028	1.511	0.168	0.164	0.160	0.555	0.077	0.629	−2.551
RMG 1087	0.000	0.167	0.167	0.167	0.574	0.000	1.000	−1.427
V 04-04	1.797	0.177	0.169	0.163	0.593	0.100	0.559	−2.176
V 6183	0.786	0.172	0.171	0.170	0.602	0.037	0.807	−1.694

**Notes.**

SSI stress susceptibility index MPI mean productivity index GMPI geometric mean productivity index HMI harmonic mean index STI stress tolerance index TI tolerance index SI stress index STS stress tolerance score

**Figure 3:**
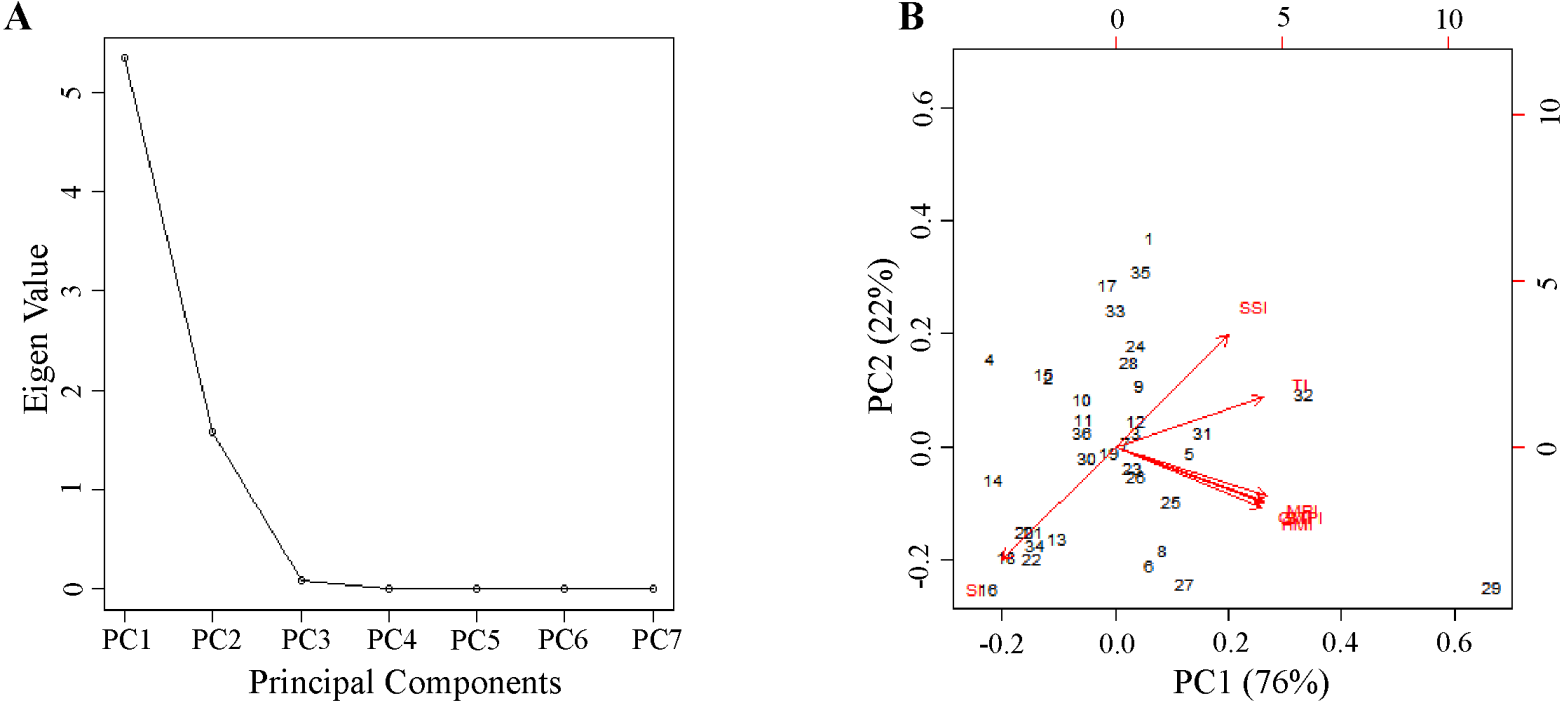
Principle component analysis of stress tolerance indexes calculated for the total dry mass of 36 genotypes. (A) Scree plot and (B) biplots of first two principal components showing the variation among the stress tolerance indexes.

**Technique 4:** This nongraphical technique is based on the STS score tabulated based on seven P deficiency tolerance indices of 36 genotypes (Table 5). Among the studied genotypes, the highest STS score was recorded by PUSA 1333 (18.873) followed by Pusa Vishal (7.250). While the lowest was recorded by IC 282094 (−6.688) followed by M1443 (−4.329). Further principal component analysis (PCA) was performed using seven P deficiency tolerance indices of 36 genotypes to identify the most contributing indices of variation. The scree plot of 36 genotypes depicted that the first two principal components (PC) showed Eigen values of more than one (Fig. 3A). According to Gatten’slower bound principle, principal components with Eigen values of less than one are ignored (Reddy et al., 2019). The first two principal components showed a variation of 76% and 22%, respectively (Table 6 and Fig. 3B). The two principal components together revealed 98% of the cumulative variation. The indices MPI and SSI explained the most percentage of variation in PC1 and PC2, respectively. The genotype PUSA 1333 showed superior performance for SSI and MPI among the 36 genotypes. Overall, three techniques except technique 2 explained that genotype PUSA 1333 followed by Pusa Vishal, PUSA 1031, and Pusa Ratna are P use efficient genotypes among the 36 genotypes used in the experiment.

**Table 6:** Principal component analysis of phosphorus deficiency tolerance indices calculated for 36 mungbean genotypes grown under normal and low P conditions.

Indexes	PC1	PC2
SSI	0.31	0.56
MPI	0.41	−0.25
GMPI	0.40	−0.28
HMI	0.40	−0.31
STI	0.40	−0.27
TI	0.40	0.25
SI	−0.31	−0.56
Eigen values	5.34	1.57
% Variance	0.76	0.22
Cumulative % Variance	0.76	0.98

**Notes.**

SSI stress susceptibility index MPI mean productivity index GMPI geometric mean productivity index HMI harmonic mean index STI stress tolerance index TI tolerance index SI stress index PC1 principal component 1 PC2 principal component 2

## Discussion

Phosphorus (P) is a major growth-limiting factor and its deficiency will severely affect seedling growth (Singh et al., 2003) and seed maturity and development at later stages (Havlin et al., 2005) in crop plants. Further, to advance the PUE improvement, it is essential to concentrate on the enhancement of both uptake and utilization efficiencies in crop plants (Weih, Hamner & Pourazari, 2018). In the present study, the screening of 36 genotypes for biomass, P uptake, and utilization efficiency showed that the mean values of RDM, RSR, and PUtE were significantly higher under the LP condition compared to the NP condition. Under low P conditions, the distribution of dry mass will increase to roots and restricts to shoot portion, thereby it favors the higher root dry mass and root to shoot ratio in crop plants (Gan et al., 2002). Thus higher root to shoot ratio under low P condition is an adaptive strategy for increasing P acquisition compared to adequate P condition (Kim & Li, 2016). The genotypes with higher root length and root hair density enhanced P uptake significantly under low P conditions (Liu et al., 2017). The higher values of PUtE under LP conditions were attributed to high values of TPU for studied genotypes. The presented results are in good harmony with the earlier reports of PUtE in rice (Wissuwa et al., 2015) and wheat (Yuan et al., 2017). The higher P utilization efficiency results from higher biomass produced per unit P uptake and P harvest index (Baligar & Fageria, 1997).

In the present investigation, the significant interaction observed between genotypes and P treatment explained that the given P treatment significantly affected the studied genotypes for the measured biomass traits, P uptake, and utilization efficiency. Further, the study reported the presence of significant variation among the genotypes under the P limiting condition. The presence of significant genetic variation coupled with high heritability is the requirement for genetic gain in selection. The study showed significant and positive correlations between TPU and biomass traits. This attribute might be due to higher cytokinins production, thereby responsible for higher biomass partitioning (Blum et al., 2014). In cotton, total P uptake showed a positive and significant correlation with root and shoot dry mass of plants (Gill et al., 2005). In addition, P uptake is also having a positive effect on leaf photosynthetic and transpiration rates (Thuynsma et al., 2016). Singh et al. (2013) observed that photosynthetic traits will be significantly affected by P deficiency and thereby reduction in plant growth in cotton. The effect of leaf photosynthetic traits under low P conditions could be studied to understand its role in P transport to root surface, thereby enhancement of P acquisition.

The gain in plant breeding program mainly confides in the selection of efficient genotypes in stress and non-stress situations. Further categorization of genotypes is a prerequisite for the improvement of PUE in future breeding ventures. Several methods and traits were used for the categorization and selection of efficient genotypes for PUE in wheat (Gill et al., 2004; Kosar et al., 2003; Gunes et al., 2006) and brassica (Aziz et al., 2011). In this study, four methods were followed for the selection of P efficient genotypes among 36 genotypes. Based on the traits, RDM SDM, RSR, TPU, and PUtE, the genotypes were classified into efficient, medium, and inefficient types. A similar type of categorization was used for identifying the efficient genotypes in wheat (Bilal et al., 2018) and brassica (Aziz et al., 2011). However, the efficient genotype under LP condition could not able to produce a similar type of performance under adequate P condition (Manske et al., 2000; Hammond & White, 2008). The genotypes showing better performance at different P levels are well adaptable to the soils having varied P conditions (Bilal et al., 2018). Therefore, categorization of genotypes under both LP and NP conditions is required. We categorized genotypes under both LP and NP conditions and also the scores of each genotype were summed to get the cumulative score of the respective genotype. The genotype PUSA 1333 showed the higher root, shoot biomass, and P uptake with a cumulative score of 27 out of 30 combining under both control and stress conditions. Whereas the genotypes IC 282094 and M1443 recorded the lowest score of 15 out of 30 combining under both P conditions. The poor performance of genotypes is mainly due to the low root, shoot biomass production, and total P uptake of genotypes under normal and low P conditions. The performance of genotypes justified the argument of the linear relationship between biomass and P uptake efficiency traits.

As per the categorization method proposed by Fageria (1993) and Bilal et al. (2018), the mungbean genotypes were categorized into four types *i.e.,* ER, ENR, IR, and INR based on P efficiency and responsiveness. The most efficient genotypes PUSA 1333 and Pusa Ratna were categorized under the ENR group under both P conditions. The genotypes ML 1666 and V6183 grouped in ER category under LP were placed in INR under NP condition. This further suggests the importance of categorization at both normal and low P levels. The genotypes grouped under ER category were well adapted to the soils with varying P levels. Whereas the genotypes classified under the ENR group could be successfully grown on P-impoverished soils. The genotypes of the IR category may be used in the crossing program for incorporating P-responsive traits. Whereas the genotypes of the INR category have no significant role in the PUE improvement program (Abbas et al., 2018). This categorization method enables the selection of genotypes suitable for a wide range of cultivation at various P levels (Irfan et al., 2020). However, this method is mainly based on population mean only. So it is having a very narrow range between efficient or inefficient and responsive or nonresponsive types (Abbas et al., 2016). For example, the genotypes M 512, ML 818, Muskan, and RMG 1028 under NP condition and the genotypes M 1316, M 961, MH 810, Muskan, and PUSA 1132 under LP condition were positioned close to the borderline of efficient and inefficient groups. Therefore, the genotypes having small divergence from the population mean are difficult to classify under efficient or inefficient and responsive or nonresponsive categories. Sothis techniqueis not suitable for studying and categorization of genotypes on large scale (Bilal et al., 2018; Irfan et al., 2020).

In another classification of Irfan et al. (2020), the genotypes were categorized into nine groups *i.e.,* LDM-HP, LDM-MP, LDM-LP, MDM-HP, MDM-MP, MDM-LP, HDM-HP, HDM-MP, and HDM-LP by developing a graph with a representation of TDM and TPU on x and *y*-axis respectively, under both NP and LP situations. This categorization method can distinguish the small differences among the genotypes by forming the nine groups (Irfan, Shah & Abbas, 2017). However, this system is more applicable to the categorization of genotypes at low P levels (Bilal et al., 2018). In the present study, the efficient genotype PUSA 1333 with high TPU and TDM grouped in HDM-HP under both NP and LP situations. The genotypes of HDM-HP are efficient in P uptake and its utilization for biomass production indicates the capability of the genotype to produce more biomass under varied P regimes (Gill et al., 2004). The genotype IC 282094 was categorized in LDM-LP and LDM-MP groups under NP and LP conditions, respectively. The genotypes of LDM-LP are least efficient in both P uptake and its utilization in biomass production. Whereas the genotypes of the LDM-MP group are efficient in P uptake but poor in their utilization for biomass production (Aziz et al., 2011). Thus three-way categorization of genotypes viz., low, medium, and high allows the detection of significant differences between high, low groups and gives maximum space for medium-type genotypes. Such differences clearly explain the adaptability of genotypes over the diverse P regimes and afford the genetic basis for the implementation of PUE improvement in breeding programs.

Thiry et al. (2016) and Grzesiak et al. (2019) classified genotypes based on stress tolerance indices estimated for the dry mass of genotypes under control and stress conditions. In the current study, the P deficiency tolerance indices of all genotypes were calculated based on TDM under both NP and LP conditions. The indices SSI, TI, and SI are susceptibility indices showing a negative relationship with yield/biomass and tend to differentiate the stress-tolerant and susceptible genotypes (Sareen, Tyagi & Sharma, 2012). Whereas the indices MPI, GMPI, and STI are tolerance indices showing a positive relationship with yield/biomass and can identify genotypes with high average yield/biomass and stress tolerance (Khodarahmpour et al., 2011). In the present study, based on PCA analysis, it is clear that two indices MPI and SSI were able to explain the most percent of the variation among the studied indices. Mohammadi, Karimizadeh & Abdipour (2011) reported that GMPI, MPI, and STI are the most recommended indices to identify the stress-tolerant genotypes under both control and stress conditions. In contrast, none of these tolerance and susceptible indices could identify the stress-tolerant genotypes with high yield/biomass under control and stress conditions (Khayatnezhad et al., 2010). Therefore, Thiry et al. (2016) suggest that a combination of the susceptible and tolerance indices will provide a useful criterion to identify the stress tolerance genotypes. The genotype PUSA 1333 in the present study showed the highest STS score indicating the high efficiency of the genotype under varied P conditions. The categorization of genotypes based on stress tolerance indices was used for the selection of drought-tolerant genotypes in wheat (Grzesiak et al., 2019), pearl millet, and sorghum (Negarestani et al., 2019). This new method of selection provides a strong criterion for categorizing the genotypes with high productivity, resilience, and clear visualization of contrast genotypes for biomass under stress conditions. This study provides new insights into the selection of genotypes as well as a better understanding of the genotype response under phosphorus stress conditions.

## Conclusions

Categorization of existing germplasm will be critical for the identification and development of more P deficiency tolerant genotypes in the mungbean breeding program. The study showed significant variation among genotypes for RDM, SDM, TDM, PC, TPU, and PUtE traits under normal and low P conditions. The presence of significant interaction among genotypes and P treatments can be exploited to develop P efficient genotypes. The P efficiency of genotypes varies with the parameters and methods used for the categorization of genotypes under control and stress conditions. The categorization of 36 genotypes by using the methods described in the study revealed that initial grouping into three classes based on efficiency followed by distribution into nine groups is the best strategy to study the minor discrepancy in the P efficiency of genotypes. In addition, the categorization based on stress tolerance score is the optimal method to visualize the contrast genotypes in terms of biomass production and resilience under low P conditions. The efficient genotype PUSA 1333 recorded better performance for dry mass production and P uptake under normal and low P conditions. Besides, the traits RDM, SDM, TDM, TPU, and PUtE were important in the categorization of genotypes for PUE in mungbean.However, the tested genotypes should be further evaluated for adult stage traits under field conditions. Overall, the results of the study could be used for the improvement of genotypes with P efficiency, which will reduce the dependency on the use of phosphatic fertilizers.

##  Supplemental Information

10.7717/peerj.12156/supp-1 Supplemental Information 1 Details of 36 mungbean genotypes used in the study

10.7717/peerj.12156/supp-2 Supplemental Information 2 Raw data of 36 genotypes

10.7717/peerj.12156/supp-3 Supplemental Information 3 Technique 1 LP

10.7717/peerj.12156/supp-4 Supplemental Information 4 Technique 1 NP

10.7717/peerj.12156/supp-5 Supplemental Information 5 Technique 2 LP

10.7717/peerj.12156/supp-6 Supplemental Information 6 Technique 2 NP

10.7717/peerj.12156/supp-7 Supplemental Information 7 Technique 3 LP

10.7717/peerj.12156/supp-8 Supplemental Information 8 Technique 3 NP

10.7717/peerj.12156/supp-9 Supplemental Information 9 Technique 4 indexes

10.7717/peerj.12156/supp-10 Supplemental Information 10 Technique 4

## Additional Information and Declarations

### Data Availability

The following information was supplied regarding data availability: The details of 36 mungbean genotypes used in the study are available in the Supplemental Files.
