# Supplementary material for: Comparison of different selection traits for identification of phosphorus use efficient lines in mungbean
Source: PeerJ. 2021 Oct 8;9:e12156. doi: 10.7717/peerj.12156 (PMC8504459; doi:10.7717/peerj.12156)
Supplement: Supplemental Information 1 [file peerj-09-12156-s001.doc]

**S1 Table. Details of 36 mungbean genotypes used in the study**

| **S. No** | **Genotype** | **Type** | **Source/ Origin** |
| --- | --- | --- | --- |
| 1 | EC 520029 | GL | Asian Vegetable Research and Development Centre, Thailand |
| 2 | EC 550851 | GL | Asian Vegetable Research and Development Centre, Thailand |
| 3 | GANGA 1 | RV | ARS Durgapura, Rajasthan, India |
| 4 | IC 282094 | GL | National Bureau of Plant Genetic Resources, India |
| 5 | IPM 02-17 | ABL | Indian Institute of Pulses Research, India |
| 6 | IPM 02-3 | RV | Indian Institute of Pulses Research, India |
| 7 | IPM 205-4 | ABL | Indian Institute of Pulses Research, India |
| 8 | KM 16-69 | ABL | AICRP, MULLaRP, India |
| 9 | KM 16-80 | ABL | AICRP, MULLaRP, India |
| 10 | LGG 460 | RV | Acharya N G Ranga Agricultural University, India |
| 11 | M 1032 | GL | Asian Vegetable Research and Development Centre, Taiwan |
| 12 | M 1129 | GL | Asian Vegetable Research and Development Centre, Taiwan |
| 13 | M 1209 | GL | Asian Vegetable Research and Development Centre, Taiwan |
| 14 | M 1316 | GL | Asian Vegetable Research and Development Centre, Taiwan |
| 15 | M 1443 | GL | Punjab Agricultural University, India |
| 16 | M 209 | GL | Punjab Agricultural University, India |
| 17 | M 512 | GL | Punjab Agricultural University, India |
| 18 | M 961 | GL | Asian Vegetable Research and Development Centre, Taiwan |
| 19 | MH 810 | RV | CCS Haryana Agricultural University, India |
| 20 | MH 934 | ABL | CCS Haryana Agricultural University, India |
| 21 | ML 1451 | GL | Punjab Agricultural University, India |
| 22 | ML 1666 | ABL | Punjab Agricultural University, India |
| 23 | ML 818 | RV | Punjab Agricultural University, India |
| 24 | MUSKAN | RV | CCS Haryana Agricultural University, India |
| 25 | PDM 139 | RV | Indian Institute of Pulses Research, India |
| 26 | PLM 167 | GL | Asian Vegetable Research and Development Centre, Thailand |
| 27 | PUSA 1031 | ABL | Indian Agricultural Research Institute, India |
| 28 | PUSA 1132 | ABL | Indian Agricultural Research Institute, India |
| 29 | PUSA 1333 | ABL | Indian Agricultural Research Institute, India |
| 30 | Pusa Baisakhi | RV | Indian Agricultural Research Institute, India |
| 31 | Pusa Ratna | RV | Indian Agricultural Research Institute, India |
| 32 | Pusa Vishal | RV | Indian Agricultural Research Institute, India |
| 33 | RMG 1028 | RV | ARS Durgapura, Rajasthan, India |
| 34 | RMG 1087 | RV | ARS Durgapura, Rajasthan, India |
| 35 | V 04-04 | GL | Asian Vegetable Research and Development Centre, Thailand |
| 36 | V 6183 | ABL | Asian Vegetable Research and Development Centre, Thailand |

RV, released variety; ABL, advanced breeding line; GL, germplasm line
